# Stationary flow driven by non-sinusoidal time-periodic pressure gradients in wavy-walled channels

**DOI:** 10.1016/j.apm.2023.06.013

**Published:** 2023-06-17

**Authors:** J. Alaminos-Quesada, C. Gutiérrez-Montes, W. Coenen, A.L. Sánchez

**Affiliations:** a Department of Mechanical and Aerospace Engineering, University of California San Diego, La Jolla, 92093-0411, California, USA; b Department of Mechanical and Mining Engineering, University of Jaén, 23071, Spain; c Andalusian Institute for Earth System Research, University of Jaén, Campus de las Lagunillas, 23071 Jaén, Spain; d Grupo de Mecánica de Fluidos, Universidad Carlos III de Madrid, Leganés, 28911, Spain

**Keywords:** Steady streaming, Oscillatory flow, Non-sinusoidal pressure

## Abstract

The classical problem of secondary flow driven by a sinusoidally varying pressure gradient is extended here to address periodic pressure gradients of complex waveform, which are present in many oscillatory physiological flows. A slender two-dimensional wavy-walled channel is selected as a canonical model problem. Following standard steady-streaming analyses, valid for small values of the ratio ε of the stroke length of the pulsatile motion to the channel wavelength, the spatially periodic flow is described in terms of power-law expansions of ε, with the Womersley number assumed to be of order unity. The solution found at leading order involves a time-periodic velocity with a zero time-averaged value at any given point. As in the case of a sinusoidal pressure gradient, effects of inertia enter at the following order to induce a steady flow in the form of recirculating vortices with zero net flow rate. An improved two-term asymptotic description of this secondary flow is sought by carrying the analysis to the following order. It is found that, when the pressure gradient has a waveform with multiple harmonics, the resulting velocity corrections display a nonzero flow rate, not present in the single-frequency case, which enables stationary convective transport along the channel. Direct numerical simulations for values of ε of order unity are used to investigate effects of inertia and delineate the range of validity of the asymptotic limit ε≪1. The comparisons of the time-averaged velocity obtained numerically with the two-term asymptotic description reveals that the latter remains remarkably accurate for values of ε exceeding 0.5. As an illustrative example, the results of the model problem are used to investigate the cerebrospinal-fluid flow driven along the spinal canal by the cardiac and respiratory cycles, characterized by markedly non-sinusoidal waveforms.

## 1. Introduction

The interaction of an oscillating stream with no-slip boundaries gives rise to a time-averaged steady-streaming motion [1]. The vast majority of studies dealing with such flows employ a sinusoidal signal to generate the primary oscillating flow. Thus, most investigations of flow over an oscillating solid body immersed in a stagnant fluid [2,3] or of flow over a fixed solid body placed in an oscillatory stream [4] assume sinusoidal oscillations, while for wall-bounded flows either the driving pressure gradient [5], the prescribed stroke volume [6], or the wall velocity [7] are assumed to vary sinusoidally in time. For geometrical configurations with streamwise symmetry, including circular cylinders [4] wavy-walled channels [8] and pipes [9], the resulting steady streaming develops in the form of recirculating vortices exhibiting symmetric closed streamlines. An important consequence is that a sinusoidal pressure gradient acting on a wall-bounded flow having streamwise symmetry, such as a wavy-walled channel [10,11], is unable to generate a steady streamwise flow rate.

Most oscillatory physiological flows driven by the cardiac and respiratory cycles display a periodic time dependence with multiple harmonics. For example, the intracranial pressure fluctuation driving the motion of cerebrospinal fluid (CSF) in the spinal canal has a waveform that includes three different peaks associated with the cardiac cycle, whose amplitudes decrease in a stepwise manner in a healthy individual [12]. The flow induced in the canal has been shown to have a stead-ysteaming component [13] that contributes fundamentally to the Lagrangian transport rate [14]. The presence of anatomical features obstructing the flow in the canal, including nerve roots and ligaments, has been shown to increase the magnitude of the resulting steady-streaming velocity [15]. Previous analytical investigations addressing this aspect of the CSF motion have employed as a canonical flow configuration a wavy-walled channel, with the associated wavelength representing the intervertebral distance [10,11,16]. These analyses, assuming a sinusoidally varying pressure gradient, showed that steady streaming occurs in the form of closed recirculating vortices confined to the channel cells, with no streamwise connectivity. Although it is unclear how a pressure gradient of complex waveform may change this symmetric recirculating flow pattern, it is worth recalling that the early analyses of secondary flows induced by cylinders and spheres undergoing non-sinusoidal oscillations [17–19] revealed the appearance of a small longitudinal flow rate aligned with the oscillation axis, not present in the sinusoidal case. Clearly, in wall-bounded flows the possible emergence of a net flow rate when the fluid is subject to a pressure gradient with complex waveform could have a large impact on the resulting longitudinal transport rate. This aspect of the problem is to be investigated below using the wavy-walled channel as illustrative canonical configuration, thereby extending the previous studies pertaining to a pure sinusoidal waveform [10,11,16].

The analysis considers the flow induced by a general time-periodic pressure difference with zero mean, for values of the oscillation period on the order of the viscous time across the canal. An asymptotic analysis is performed for values of the stroke length much smaller than the channel wavelength, a familiar limit [1] in which the problem is linear at leading order, resulting in an oscillatory velocity with zero mean that can be expressed in Fourier form [20]. The first-order velocity corrections contain a stationary component in the form of closed symmetric recirculating vortices with no net flow rate. Symmetry breaking leading to a stationary streamwise flow rate arises at the following order in the expansion for small stroke lengths as a result of the interactions between different modes in the Fourier expansions. This finding is consistent with the previous analyses of the flow induced by oscillating circular cylinders and spheres [17–19].

The flow velocity predicted by the asymptotic analysis, including a small net flow rate varying quadratically with the stroke length, is to be compared below with results of direct numerical simulations. The comparisons reveal that the accuracy of the asymptotic results extends beyond the expected range of validity, with the predicted mean flow rate showing good agreement with the numerical results for values of the stroke length exceeding half of the channel wavelength. The influence of the waveform on the resulting net flow rate is illustrated by considering the pressure differences that characterize the cardiac and respiratory motion along the spinal canal, with the induced streamwise flow rate found to be more prominent in the former case.

## 2. Problem formulation

**Fig. 1.**
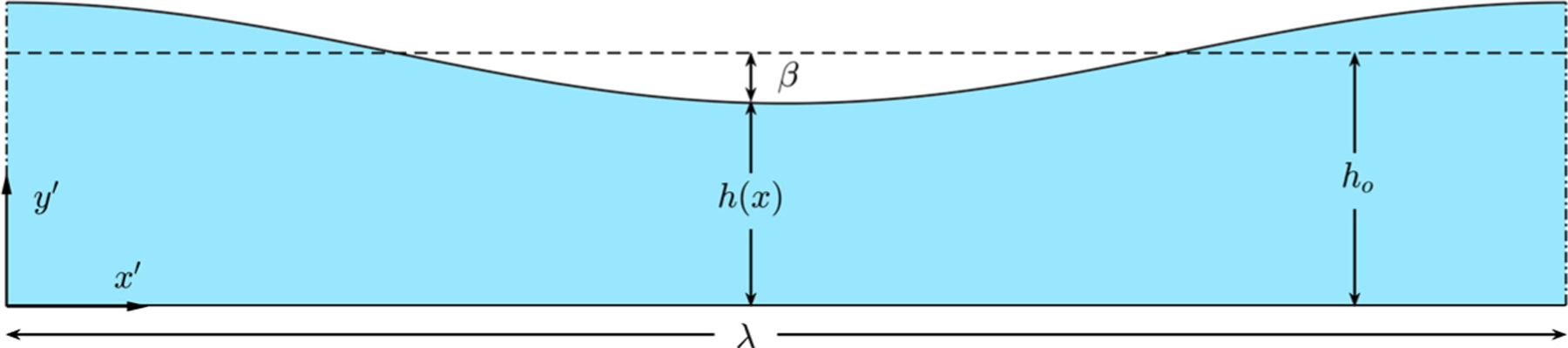
Schematic of the wavy channel of wave length λ, with an average channel width $h_{0}$ and a relative amplitude of the wall undulation β.

Consider a wavy channel of average gap size $h_{0}$ filled with a Newtonian fluid of density ρ and kinematic viscosity ν. The channel is bounded by a flat surface and a wavy wall of wave length $\lambda \gg h_{0}$, so that the resulting channel width h is described by the periodic dimensionless function $h/h_{0}=H\left(x^{\prime }/\lambda \right)$, where $x^{\prime }$ is the longitudinal distance measured along the channel. Although the analysis is carried out for wavy walls of general shape, the figures plotted below as well as the accompanying direct numerical simulations correspond to $H=\left[1+\beta \text{cos}\left(2\pi x^{\prime }/\lambda \right)\right]$, where β<1 is the relative amplitude of the wall undulation (see Fig. 1). In previous steady-streaming analyses of wall-bounded flows [11], the wavy-walled channel has been reasoned to be a model for the subarachnoid space surrounding the spinal cord, for which $h_{0}≃1-4\;\text{mm}$, with the wave length λ≃2−4cm representing the distance between vertebrae, which cause canal obstructions that can be estimated to correspond to undulations in the range 0.2≲β≲0.4 [21,22].

We investigate the spatially periodic flow driven by a time-periodic pressure gradient with zero mean, resulting in a pressure difference per wavelength $\Delta p\Pi \left(\omega t^{\prime }\right)$, where Δp is the characteristic amplitude, ω is the angular frequency and $t^{\prime }$ represents the time. The 2π periodic dimensionless function $\Pi \left(\omega t^{\prime }\right)$, describing the waveform of the pressure fluctuation, is expressible in the general Fourier-expansion form $\Pi \left(\omega t^{\prime }\right)=\sum_{n=1}^{\infty }\;R\left[A_{n}\text{exp}\left(\text{i}n\omega t^{\prime }\right)\right]$, where the order-unity coefficients $A_{n}$ are complex numbers and R denotes the real part of a complex function. Since the flow is spatially periodic, it suffices to investigate the solution in the channel stretch $0\leq x^{\prime }\leq \lambda$, with the spatial pressure variation $p^{\prime }$ satisfying $p^{\prime }=0$ at $x^{\prime }=0$ and $p^{\prime }=\Delta p\Pi \left(\omega t^{\prime }\right)$ at $x^{\prime }=\lambda$. The description employs cartesian coordinates $\left(x^{\prime },y^{\prime }\right)$, with $y^{\prime }$ measured from the flat surface, and corresponding velocity components $\left(u^{\prime },v^{\prime }\right)$.

The resulting slender flow is characterized by longitudinal velocities of order $u_{c}=\Delta p/(\rho \omega \lambda )$, as follows from a balance between the local acceleration $\partial u^{\prime }/\partial t^{\prime }\sim u_{c}\omega$ and the pressure gradient $\rho^{-1}\partial p^{\prime }/\partial x^{\prime }\sim \Delta p/(\lambda \rho )$, and much smaller transverse velocities of order $v_{c}=\left(h_{0}/\lambda \right)u_{c}\ll u_{c}$, as follows from the continuity balance $\partial u^{\prime }/\partial x^{\prime }\sim \partial v^{\prime }/\partial y^{\prime }$. Introducing the dimensionless variables

(1) $$t=\omega t^{\prime },x=\frac{x^{\prime }}{\lambda },y=\frac{y^{\prime }}{h_{0}},u=\frac{u^{\prime }}{u_{c}},v=\frac{v^{\prime }}{v_{c}},p=\frac{p^{\prime }}{\Delta p}$$

reduces the conservation equations to

(2) $$\frac{\partial u}{\partial x}+\frac{\partial v}{\partial y}=0,$$

(3) $$\frac{\partial u}{\partial t}+\varepsilon \left(u\frac{\partial u}{\partial x}+v\frac{\partial u}{\partial y}\right)=-\frac{\partial p}{\partial x}+\frac{1}{\alpha^{2}}\left[{\left(\frac{h_{o}}{\lambda }\right)}^{2}\frac{\partial^{2}u}{\partial x^{2}}+\frac{\partial^{2}u}{\partial y^{2}}\right],$$

(4) $$\frac{\partial v}{\partial t}+\varepsilon \left(u\frac{\partial v}{\partial x}+v\frac{\partial v}{\partial y}\right)=-{\left(\frac{h_{o}}{\lambda }\right)}^{-2}\frac{\partial p}{\partial y}+\frac{1}{\alpha^{2}}\left[{\left(\frac{h_{o}}{\lambda }\right)}^{2}\frac{\partial^{2}v}{\partial x^{2}}+\frac{\partial^{2}v}{\partial y^{2}}\right].$$

The velocity is periodic in x, i.e. u(0,y,t)=u(1,y,t) and v(0,y,t)=v(1,y,t), and satisfies the no-slip boundary condition

(5) $$u=v=0\text{ at }\left\{\begin{array}{l} y=0\\ y=H=1+\beta \text{cos}(2\pi x) \end{array},\right.$$

whereas the pressure p is identically zero at x=0 and takes the value p=Π(t) at x=1, with

(6) $$\Pi (t)=\sum_{n=1}^{\infty }\;R\left(A_{n}{\text{e}}^{\text{i}\text{nt}}\right).$$

**Fig. 2.**
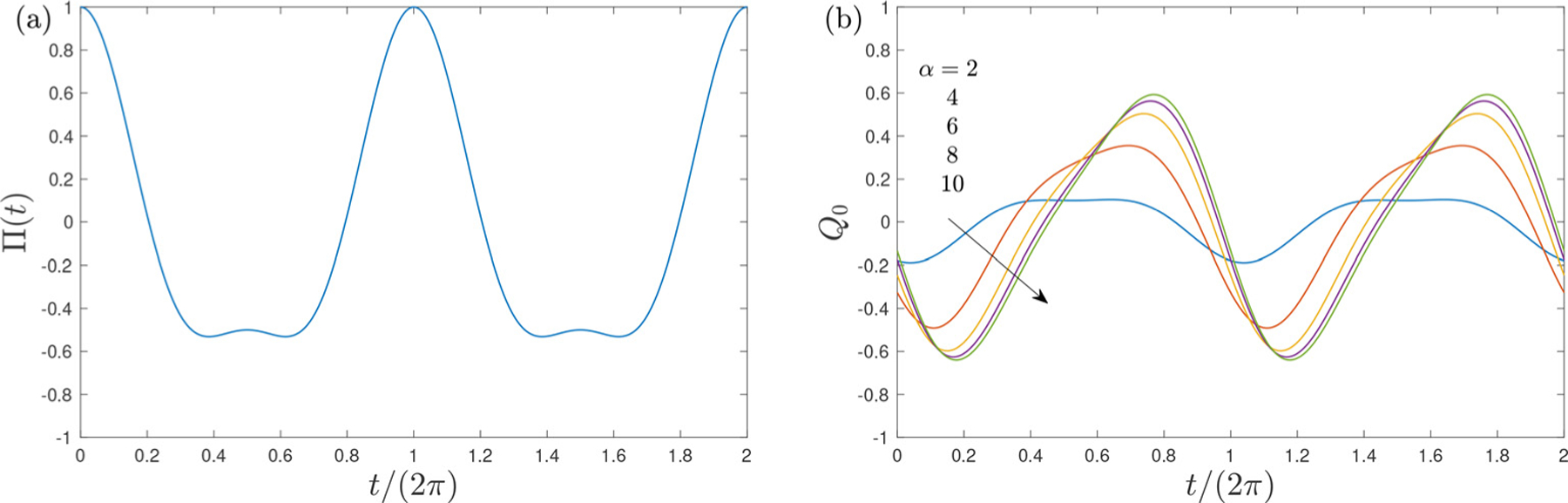
The dimensionless pressure waveform Π(r) corresponding to $A_{1}=3/4$ and $A_{2}=1/4$ (a) and the associated volumetric flow rate evaluated from (19) for β=0.4 and α=2:2:10 (b).

The sinusoidal case can be investigated by considering $A_{1}=1$ with $A_{n}=0$ for n≥2. A pressure signal of complex waveform can be obtained by combining different modes with nonzero coefficients $A_{n}$. For instance, the pressure function Π(t) corresponding to $A_{1}=3/4$ and $A_{2}=1/4$, used for some of the sample computations presented below, is shown in Fig. 2(a).

As can be seen, the solution depends on two flow parameters, namely, the Womersley number

(7) $$\alpha ={\left(\frac{\omega h_{o}^{2}}{v}\right)}^{1/2},$$

representing the ratio of the viscous time across the channel $h_{0}^{2}/v$ to the characteristic oscillation time $\omega^{-1}$, and the Strouhal number $\varepsilon^{-1}$, with

(8) $$\varepsilon =\frac{u_{c}/\omega }{\lambda },$$

representing the ratio of the stroke lengths $u_{c}/\omega$ to the channel wavelength λ. The solution exhibits an additional dependence on the slenderness ratio $h_{0}/\lambda \ll 1$.

The direct numerical simulations to be presented later employ the complete Eqs. (2)–(4). By way of contrast, the analytical development, using asymptotic expansions in powers of ε≪1, will exploit the slenderness of the flow in simplifying the equations by assuming $h_{0}/\lambda \ll \varepsilon$. Under those conditions, the slender-flow approximation, used in other analyses of steady streaming in channels [5–8,10,11,16], applies to the description of terms up to order $\varepsilon^{2}$, with (4) reducing to ∂p/∂y=0 and (3) yielding

(9) $$\frac{\partial u}{\partial t}+\varepsilon \left(u\frac{\partial u}{\partial x}+v\frac{\partial u}{\partial y}\right)=-\frac{\partial p}{\partial x}+\frac{1}{\alpha^{2}}\frac{\partial^{2}u}{\partial y^{2}},$$

to be used together with (2) as starting point in the asymptotic analysis.

A key feature of the solution concerns the resulting mean flow rate $\langle Q\rangle =\int_{0}^{H}\;\langle u\rangle \text{d}y$, where

(10) $$\langle \cdot \rangle =\frac{1}{2\pi }\int_{t}^{t+2\pi }\;\cdot \text{d}t$$

represents the time-averaging operator. While ⟨Q⟩ was previously found to be identically zero for flow driven by a sinusoidal pressure gradient in infinite wavy-walled channels [10,11,16] and also in general channels with H(0)=H(1) [23], in the nonsinusoidal case treated here the flow rate will be shown to exhibit a small steady component of order $\varepsilon^{2}$.

## 3. Asymptotic analysis for small stroke lengths

In the asymptotic limit ε≪1 the slender-flow problem can be solved by substituting the asymptotic expansions $u=u_{0}(x,y,t,\tau )+\varepsilon u_{1}(x,y,t,\tau )+\cdots ,v=v_{0}(x,y,t,\tau )+\varepsilon v_{1}(x,y,t,\tau )+\cdots$, and $p=p_{0}(x,t,\tau )+\varepsilon p_{1}(x,t,\tau )+\cdots$ into (2) and (9) and solving sequentially the problems that arise at different orders in powers of ε. The solution, determined in [16] for the canonical case Π(t)=cos⁡(t) up to terms of order ε, is extended here to order $\varepsilon^{2}$ for driving pressure differences of general waveform (6). The corresponding expansion for the mean flow rate

(11) $$\langle Q\rangle =\int_{0}^{H}\;\langle u\rangle \text{d}y=\left\langle Q_{0}\right\rangle +\varepsilon \left\langle Q_{1}\right\rangle +\varepsilon^{2}\left\langle Q_{2}\right\rangle +\cdots ,$$

with $\left\langle Q_{i}\right\rangle =\int_{0}^{H}\;\left\langle u_{i}\right\rangle \text{d}y$ for i=0,1,2,…, will be shown to give $\left\langle Q_{0}\right\rangle =0$ and $\left\langle Q_{1}\right\rangle =0$, so that the analysis needs to be carried to order $\varepsilon^{2}$ to determine the first nonzero term $\left\langle Q_{2}\right\rangle \neq 0$.

### 3.1. Leading-order solution

The linear problem that arises at zeroth order, involving the integration of

(12) $$\frac{\partial u_{0}}{\partial x}+\frac{\partial v_{0}}{\partial y}=0\text{ and }\frac{\partial u_{0}}{\partial t}=-\frac{\partial p_{0}}{\partial x}+\frac{1}{\alpha^{2}}\frac{\partial^{2}u_{0}}{\partial y^{2}}$$

with boundary conditions $u_{0}=v_{0}=0$ at $y=0,H(x),p_{0}=0$ at x=0 and $p_{0}=\Pi (t)$ at x=1, can be solved exactly to give

(13) $$u_{0}=\sum_{n=1}^{\infty }\;R\left(\frac{A_{n}}{n}\text{i}{\text{e}}^{\text{i}\text{nt}}U_{n}\right)\text{ and }v_{0}=\sum_{n=1}^{\infty }\;R\left(\frac{A_{n}}{n}\text{i}{\text{e}}^{\text{i}\text{nt}}V_{n}\right)\text{,}$$

where

(14) $$U_{n}=\frac{\text{d}P_{n}}{\;\text{d}x}G_{n}\;\text{ and }\;V_{n}=-\frac{\partial }{\partial x}\left(\frac{\text{d}P_{n}}{\;\text{d}x}\int_{0}^{y}\;\;G_{n}\;\text{d}\tilde{y}\right),$$

with the auxiliary functions

(15) $$\frac{\text{d}P_{n}}{\;\text{d}x}(x)={\left[H\left(1-\Lambda_{n}^{-1}\text{tanh}\Lambda_{n}\right)\int_{0}^{1}\;\;\frac{\text{d}x}{H\left(1-\Lambda_{n}^{-1}\text{tanh}\Lambda_{n}\right)}\right]}^{-1},$$

(16) $$G_{n}(x,y)=1-\frac{\text{cosh}\left[\Lambda_{n}(2y/H-1)\right]}{\text{cosh}\Lambda_{n}},$$

and

(17) $$\int_{0}^{y}\;G_{n}\;\text{d}\tilde{y}=y-\frac{H}{2\Lambda_{n}}\left\{\frac{\text{sinh}\left[\Lambda_{n}(2y/H-1)\right]+\text{sinh}\Lambda_{n}}{\text{cosh}\Lambda_{n}}\right\}$$

involving the complex Womersley function

(18) $$\Lambda_{n}=\frac{\sqrt{n}\alpha }{2}\frac{1+\text{i}}{\sqrt{2}}H(x)$$

evaluated with the frequency nω at the local height H(x). A dummy integration variable $\tilde{y}$ is introduced for convenience in (14). As is clear from (13), the leading-order solution has zero time-averaged velocities $\left\langle u_{0}\right\rangle =\left\langle v_{0}\right\rangle =0$. Consequently, the leading-order volumetric flow rate

(19) $$Q_{0}=\int_{0}^{H}\;u_{0}\;\text{d}y=\sum_{n=1}^{\infty }\;R\left[\frac{\text{i}{\text{e}}^{\text{i}\text{nt}}A_{n}/n}{\int_{0}^{1}\;\;{\left[H\left(1-\Lambda_{n}^{-1}\text{tanh}\Lambda_{n}\right)\right]}^{-1}\;\text{d}x}\right]$$

also has a zero mean value $\left\langle Q_{0}\right\rangle =0$. Sample flow rates $Q_{0}(t)$ computed from (19) for the pressure wave form of Fig. 2(a) are shown in Fig. 2(b).

### 3.2. First-order corrections

The solution at the following order (ε) involves the integration of

(20) $$\frac{\partial u_{1}}{\partial x}+\frac{\partial v_{1}}{\partial y}=0,$$

(21) $$\frac{\partial u_{1}}{\partial t}+\frac{\partial }{\partial x}(u_{0}^{2})+\frac{\partial }{\partial y}(u_{0}v_{0})=-\frac{\partial p_{1}}{\partial x}+\frac{1}{\alpha^{2}}\frac{\partial^{2}u_{1}}{\partial y^{2}},$$

with boundary conditions $u_{1}=v_{1}=0$ at y=0,H and $p_{1}=0$ at x=0,1. The convective acceleration corresponding to the leading-order solution, driving the velocity corrections at this order, can be expressed in the form

(22) $$\frac{\partial }{\partial x}\left(u_{0}^{2}\right)+\frac{\partial }{\partial y}\left(u_{0}v_{0}\right)=\sum_{n=-\infty }^{\infty }\;F_{n}(x,y){\text{e}}^{\text{i}\text{nt}},$$

where

(23) $$F_{n}=\overset{\infty }{\underset{m=n+1}{\sum }}\frac{A_{m}A_{m-n}^{\text{*}}}{2m\left(m-n\right)}\left[\frac{\partial }{\partial x}\left(U_{m}U_{m-n}^{\text{*}}\right)+\frac{1}{2}\frac{\partial }{\partial y}\left(U_{m}V_{m-n}^{\text{*}}+U_{m-n}^{\text{*}}V_{m}\right)\right]\;\;-\overset{n-1}{\underset{\begin{array}{l} m=1\\ n>1 \end{array}}{\sum }}\frac{A_{m}A_{n-m}}{4m\left(n-m\right)}\left[\frac{\partial }{\partial x}\left(U_{m}U_{n-m}\right)+\frac{\partial }{\partial y}\left(U_{m}V_{n-m}\right)\right]$$

for n≥0 and $F_{n}=F_{-n}^{*}$ for n<0, with the asterisk * denoting complex conjugates. Note that the second line of the above equation gives no contribution to the evaluation of $F_{0}$ and $F_{\pm 1}$. With the exception of

(24) $$F_{0}=\sum_{n=1}^{\infty }\;{\left(\frac{A_{n}A_{n}^{*}}{n}\right)}^{2}\left[\frac{1}{2}\frac{\partial }{\partial x}\left(U_{n}U_{n}^{*}\right)+\frac{1}{4}\frac{\partial }{\partial y}\left(U_{n}V_{n}^{*}+U_{n}^{*}V_{n}\right)\right],$$

which is real, all $F_{n}(x,y)$ are in general complex functions. Also of interest is that in the sinusoidal case

(25) $$F_{0}=\frac{1}{2}\frac{\partial }{\partial x}\left(U_{1}U_{1}^{*}\right)+\frac{1}{4}\frac{\partial }{\partial y}\left(U_{1}V_{1}^{*}+U_{1}^{*}V_{1}\right)$$

and

$$F_{2}=F_{-2}^{*}=-\frac{1}{4}\left[\frac{\partial }{\partial x}\left(U_{1}^{2}\right)+\frac{\partial }{\partial y}\left(U_{1}V_{1}\right)\right],$$

all other $F_{n}(x,y)$ being identically zero.

#### 3.2.1. Steady velocity corrections

The velocity corrections $u_{1}=u_{1}^{s}+u_{1}^{u}$ and $v_{1}=v_{1}^{s}+v_{1}^{u}$ can be expressed as the sum of a steady part $\left(u_{1}^{s},v_{1}^{s}\right)$, corresponding to a time-independent secondary flow, and an unsteady part $\left(u_{1}^{u},v_{1}^{u}\right)$, with the latter satisfying $\left\langle u_{1}^{u}\right\rangle =\left\langle v_{1}^{u}\right\rangle =0$. The reduced equations for the steady components follow from taking the time average of (20) and (21) to give

(27) $$\frac{\partial u_{1}^{s}}{\partial x}+\frac{\partial v_{1}^{s}}{\partial y}=0\;\text{and}\;F_{\text{0}}=-\frac{\partial p_{1}^{s}}{\partial x}+\frac{1}{\alpha^{2}}\frac{\partial^{2}u_{1}^{s}}{\partial y^{2}},$$

which can be integrated with boundary conditions $u_{1}^{s}=v_{1}^{s}=0$ at y=0,H and $p_{1}^{s}=0$ at x=0,1 to yield

(28) $$\frac{u_{1}^{s}}{\alpha^{2}}=-\frac{\text{d}p_{1}^{s}}{\text{d}x}\frac{1}{2}(H-y)y+y\int_{0}^{y}F_{0}\;\text{d}\tilde{y}-\int_{0}^{y}F_{0}\tilde{y}\;\text{d}\tilde{y}-y\int_{0}^{H}F_{0}\left(1-\frac{y}{H}\right)\text{dy,}$$

and

(29) $$\frac{v_{1}^{s}}{\alpha^{2}}=\frac{\partial }{\partial x}[\frac{\text{d}p_{1}^{s}}{\text{ d}x}\frac{y^{2}}{2}\left(\frac{H}{2}-\frac{y}{3}\right)+\frac{y^{2}}{2}\int_{y}^{H}F_{0}\left(1-\frac{\tilde{y}}{H}\right)\text{d}\tilde{y}+y\left(1-\frac{y}{2H}\right)\int_{0}^{y}F_{0}\tilde{y}\text{ d}\tilde{y}-\frac{1}{2}\int_{0}^{y}F_{0}{\tilde{y}}^{2}\text{ d}\tilde{y}],$$

where the pressure gradient can be shown to reduce to

(30) $$\frac{\text{d}p_{1}^{s}}{\;\text{d}x}=-6H^{-3}\int_{0}^{H}\;F_{0}y(H-y)\text{d}y$$

when account is taken of the condition H(0)=H(1). The steady flow rate at this order is identically zero, i.e. $\left\langle Q_{1}\right\rangle =\int_{0}^{H}\;u_{1}^{s}\;\text{d}y=0$, a result in agreement with previous findings regarding steady streaming in channels [10,11,16] and tubes [5].

Before we proceed further with the asymptotic description, it is instructive to analyze the characteristics of the streaming flow emerging at this order, described by the above Eqs. (28)–(30). Observation of (24) reveals that the steady-streaming velocities (28) and (29) result from nonlinear interactions of each Fourier mode with itself, a result encountered by Davidson and Riley [20] in their analysis of flow over oscillating cylinders. In the absence of inter-mode interactions, the associated stationary motion is just the linear superposition of the steady-streaming velocities associated with sinusoidal pressure gradients whose frequencies differ by an integer factor. Since each mode produces stationary vortices with no streamwise flow rate, their linear combination is also a periodic array of separate stationary vortices recirculating in each channel cell with no net streamwise flow rate.

**Fig. 3.**
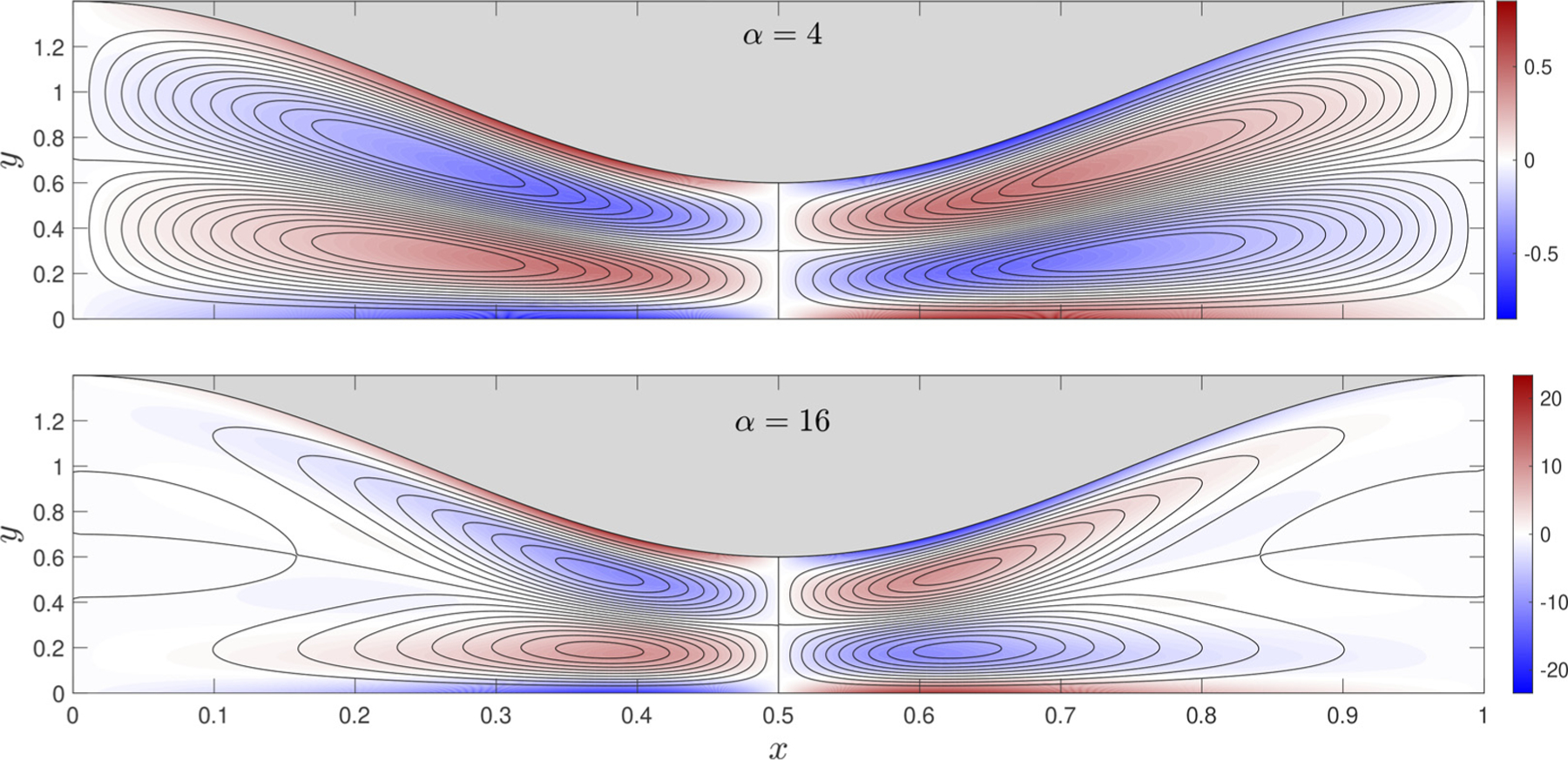
Streamlines and colour contours of vorticity corresponding to the first-order steady-streaming velocity $v_{1}^{s}$ driven by a pressure difference Π ((L)= (3/4)cos⁡(t)+(1/4)cos⁡(2t) in a canal with β=0.4 for α=4 and α=16, A fixed constant streamline spacing $\delta \psi_{1}$ is used in drawing the streamlines, with $\delta \psi_{1}=5\cdot 10^{-4}$ for α=4 and $\delta \psi_{1}=10^{-2}$ for α=16.

To illustrate this feature of the solution, the streamlines and vorticity corresponding to the non-sinusoidal pressure of Fig. 2(a) are plotted in Fig. 3 for β=0.4 and for two values of the Womersley number, α=4 and α=16. As can be seen, the flow structure, similar to that obtained in previous channel-flow analyses [11,16], exhibits recirculating vortices separated by the vertical streamlines x=0,0.5,1. For α=4 the flow in each half cell exhibits two counter-rotating vortices, whereas for α=16 the flow develops an additional, much weaker vortex, located near the section with largest width. The streamlines are plotted using fixed increments $\delta \psi_{1}$ of the streamfunction $\psi_{1}$, defined in the usual way (i.e. by integration of $\partial \psi_{1}/\partial y=u_{1}^{s}$ and $\partial \psi_{1}/\partial x=-v_{1}^{s}$ with $\psi_{1}=0$ along the wall), so that the interline spacing provides a measure of the local velocity. To further quantify the motion, color contours are used to represent the associated vorticity $\Omega ={\left(h_{0}/\lambda \right)}^{2}\partial v/\partial x-\partial u/\partial y$, which reduces to Ω=−∂u/∂y in the slender flow approximation employed here.

As previously mentioned, the streaming flow at this order has a zero flow rate $\left\langle Q_{1}\right\rangle =\int_{0}^{H}\;u_{1}^{s}\;\text{d}y=0$, consistent with the recirculating patterns depicted in Fig. 3. A nonzero flow rate will be seen to emerge at the following order in the description, to be presented below. Similar higher-order steady-streaming analyses have been performed for flows over oscillatory circular cylinders and spheres [17–19], for which the complex waveform breaks the fore-and-aft symmetry of the flow.

#### 3.2.2. Unsteady velocity corrections

The unsteady component of the first-order velocity corrections can be expressed in the general form

(31) $$u_{1}^{u}=\overset{\infty }{\underset{\begin{array}{c} n=-\infty \\ n\neq 0 \end{array}}{\sum }}ℜ\left(\frac{\text{i}}{\left|n\right|}{\text{e}}^{\text{i}nt}{\hat{U}}_{n}\right)\text{ and }v_{1}^{u}=\overset{\infty }{\underset{\begin{array}{c} n=-\infty \\ n\neq 0 \end{array}}{\sum }}ℜ\left(\frac{\text{i}}{\left|n\right|}{\text{e}}^{\text{i}nt}{\hat{V}}_{n}\right)$$

where the complex functions ${\overset{ˆ}{U}}_{n}(x,y)$ and ${\overset{ˆ}{V}}_{n}(x,y)$ satisfy

(32) $$\frac{\partial {\hat{U}}_{n}}{\partial x}+\frac{\partial {\hat{V}}_{n}}{\partial y}=0\text{ and }\frac{\text{i}}{\alpha^{2}n}\frac{\partial^{2}{\hat{U}}_{n}}{\partial y^{2}}+{\hat{U}}_{n}=\frac{\text{d}{\hat{P}}_{n}}{\text{ d}x}+F_{n}$$

with boundary conditions ${\overset{ˆ}{U}}_{n}={\overset{ˆ}{V}}_{n}=0$ at y=0,H and ${\overset{ˆ}{P}}_{n}=0$ at x=0,1. The problem can be integrated to yield

(33) $${\hat{U}}_{n}=\frac{\text{d}{\hat{P}}_{n}}{\text{ d}x}G_{n}+T_{n}\text{ and }{\hat{V}}_{n}=-\frac{\partial }{\partial x}\left[\frac{\text{d}{\hat{P}}_{n}}{\text{ d}x}\int_{0}^{y}G_{n}\text{ d}\hat{y}+\int_{0}^{y}T_{n}\text{ d}\hat{y}\right]\text{,}$$

where the pressure gradient can be seen to reduce to

(34) $$\frac{\text{d}{\hat{P}}_{n}}{\text{ d}x}=\frac{\int_{0}^{H}F_{n}G_{n}\text{ d}y}{H\left(\Lambda_{n}^{-1}{\text{tanhΛ}}_{n}-1\right)}$$

when account is taken of the condition H(0)=H(1). The functions $G_{n}$ and $\int_{0}^{y}\;G_{n}\;\text{d}\tilde{y}$ appearing in (33) are defined in (16) and (17), with |n| used to evaluate these expressions when n<0. The additional auxiliary functions $T_{n}$ and $\int_{0}^{y}\;T_{n}\;\text{d}\tilde{y}$ can be evaluated from

(35) $$T_{n}=\frac{2\Lambda_{n}}{H}\{\text{cosh}\left(2\Lambda_{n}\frac{y}{H}\right)\int_{0}^{y}F_{n}\text{sinh}\left(2\Lambda_{n}\frac{y}{H}\right)\text{d}\tilde{y}\;+\text{sinh}\left(2\Lambda_{n}\frac{y}{H}\right)\left[\frac{\int_{0}^{H}F_{n}\text{sinh}\left[2\Lambda_{n}\left(1-\frac{y}{H}\right)\right]\text{d}y}{\text{sinh}\left(2\Lambda_{n}\right)}-\int_{0}^{y}F_{n}\text{cosh}\left(2\Lambda_{n}\frac{y}{H}\right)\text{d}\tilde{y}\right]\}$$

and

(36) $$\int_{0}^{y}T_{n}\text{ d}\tilde{y}=\int_{0}^{y}F_{n}\text{ d}\tilde{y}+\frac{\text{cosh}\left(2\Lambda_{n}\frac{y}{H}\right)-1}{\text{sinh}\left(2\Lambda_{n}\right)}\int_{0}^{H}F_{n}\text{sinh}\left[2\Lambda_{n}\left(1-\frac{y}{H}\right)\right]\text{d}y+\text{sinh}\left(2\Lambda_{n}\frac{y}{H}\right)\int_{0}^{y}F_{n}\text{sinh}\left(2\Lambda_{n}\frac{y}{H}\right)\text{d}\tilde{y}-\text{cosh}\left(2\Lambda_{n}\frac{y}{H}\right)\int_{0}^{y}F_{n}\text{cosh}\left(2\Lambda_{n}\frac{y}{H}\right)\text{d}\tilde{y},$$

which completes the description of the first-order corrections. It is worth noting that, in the sinusoidal case, the only nonzero terms in (31) are n=±2, so that the unsteady component of the velocity correction can be reduced with use of (26) to

(37) $$u_{1}^{u}=-I\left({\text{e}}^{2\text{i}t}{\overset{ˆ}{U}}_{2}\right)\text{ and }v_{1}^{u}=-I\left({\text{e}}^{2\text{i}t}{\overset{ˆ}{V}}_{2}\right),$$

where I denotes the imaginary part of a complex function.

### 3.3. Higher-order corrections

Just like the first-order corrections, the velocity corrections $\left(u_{2},v_{2}\right)$ emerging at order $\varepsilon^{2}$ include a time-independent component $\left(u_{2}^{s},v_{2}^{s}\right)=\left(\left\langle u_{2}\right\rangle ,\left\langle v_{2}\right\rangle \right)$, which can be obtained by time averaging the corresponding conservation equations to give

(38) $$\frac{\partial u_{2}^{s}}{\partial x}+\frac{\partial v_{2}^{s}}{\partial y}=0\;\text{and}\;ℱ\;=\;-\frac{\partial p_{2}^{s}}{\partial x}+\frac{1}{\alpha^{2}}\frac{\partial^{2}u_{2}^{s}}{\partial y^{2}},$$

to be integrated with boundary conditions $u_{2}^{s}=v_{2}^{s}=0$ at y=0,H and $p_{2}^{s}=0$ at x=0,1. The forcing term

(39) $$ℱ(x,y)=2\frac{\partial }{\partial x}\left\langle u_{0}u_{1}^{u}\right\rangle +\frac{\partial }{\partial y}\left\langle u_{0}v_{1}^{u}+v_{0}u_{1}^{u}\right\rangle$$

arises from time-averaging the convective acceleration at this order. In the sinusoidal case one finds ℱ=0, because $\left(u_{0},v_{0}\right)\propto {\text{e}}^{\text{i}t}$ and $\left(u_{1}^{u},v_{1}^{u}\right)\propto {\text{e}}^{2\text{i}t}$, so that the solution to (38) reduces to $u_{2}^{s}=v_{2}^{s}=0$. On the other hand, for a non-sinusoidal periodic waveform, the forcing term can be expressed in the form

(40) $$ℱ=R\left\{\sum_{n=1}^{\infty }\;\;\frac{A_{n}^{*}}{n^{2}}\left[2\frac{\partial }{\partial x}\left(U_{n}^{*}{\overset{ˆ}{U}}_{n}\right)+\frac{\partial }{\partial y}\left(U_{n}^{*}{\overset{ˆ}{V}}_{n}+V_{n}^{*}{\overset{ˆ}{U}}_{n}\right)\right]\right\},$$

which can be evaluated by substituting the velocity components shown in (14) and (33).

Except for the forcing function in the momentum equation, the problem defined above for $\left(u_{2}^{s},v_{2}^{s}\right)$ is identical to that determining $\left(u_{1}^{S},v_{1}^{S}\right)$, so that the solution can be trivially obtained by replacing $F_{0}$ with ℱ in (28) and (29) to give

(41) $$\frac{u_{2}^{s}}{\alpha^{2}}=-\frac{\text{d}p_{2}^{s}}{\text{d}x}\frac{1}{2}(H-y)y+y\int_{0}^{y}ℱ\;\text{d}\tilde{y}-\int_{0}^{y}ℱ\tilde{y}\;\text{d}\tilde{y}-y\int_{0}^{y}ℱ\left(1-\frac{y}{H}\right)\text{d}y,$$

and

(42) $$\frac{v_{2}^{s}}{\alpha^{2}}=\frac{\partial }{\partial x}\left[\frac{\text{d}p_{2}^{s}}{\text{d}x}\frac{y^{2}}{2}\left(\frac{H}{2}-\frac{y}{3}\right)+\frac{y^{2}}{2}\int_{0}^{H}ℱ\left(1-\frac{\tilde{y}}{H}\right)\text{d}\tilde{y}+y\left(1-\frac{y}{2H}\right)\int_{0}^{y}ℱ\tilde{y}\;\text{d}\tilde{y}-\frac{1}{2}\int_{0}^{y}ℱ\tilde{y}{\;}^{2}\text{d}\tilde{y}\right],$$

where the pressure gradient is given in this case by

(43) $$\frac{\text{d}p_{2}^{s}}{\;\text{d}x}=\frac{6}{H^{3}}\left[\frac{\int_{0}^{1}\;\;H^{-3}\left[\int_{0}^{H}\;\;y(H-y)ℱ\text{d}y\right]\text{d}x}{\int_{0}^{1}\;\;H^{-3}\;\text{d}x}-\int_{0}^{H}\;\;ℱy(H-y)\text{d}y\right].$$

The value of $u_{2}^{s}$ given in (41) can be used to compute $\left\langle Q_{2}\right\rangle =\int_{0}^{H}\;u_{2}^{s}\;\text{d}y$, which can be substituted into (11) to give

(44) $$\frac{\left\langle Q\right\rangle }{\varepsilon^{2}\alpha^{2}}=-\frac{1}{2}\frac{\int_{0}^{1}\;\;H^{-3}\left[\int_{0}^{H}\;\;y(H-y)ℱ\text{d}y\right]\text{d}x}{\int_{0}^{1}\;\;H^{-3}\;\text{d}x},$$

describing the mean flow rate with small relative errors of order ε. As can be seen in (44), the value of ⟨Q⟩ is directly proportional to $\varepsilon^{2}$ and $\alpha^{2}$, with the Womersley number entering also through the forcing term ℱ.

## 4. Illustrative results

### 4.1. Second-order steady streaming

**Fig. 4.**
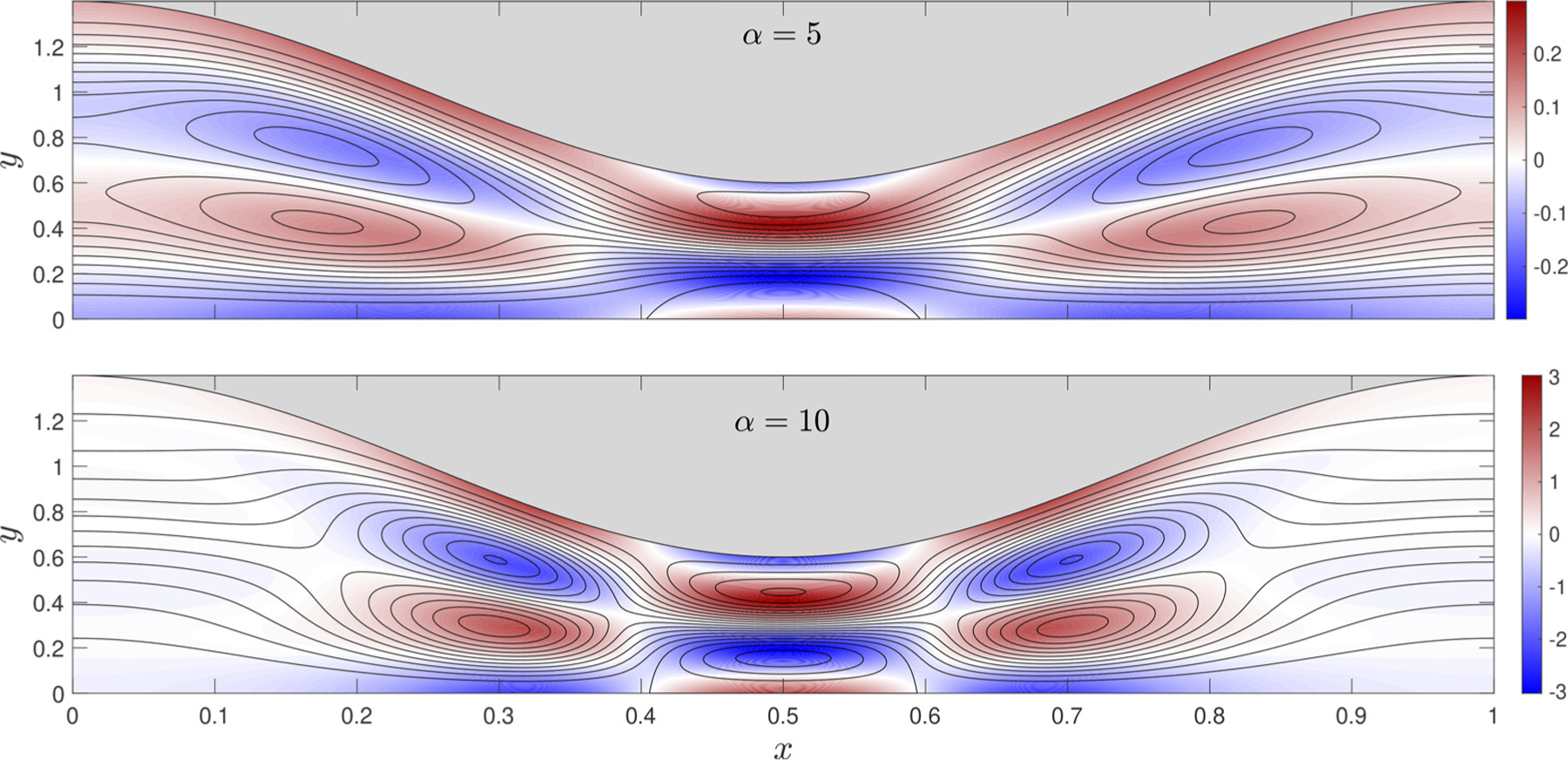
Streamlines and colour contours of vorticity corresponding to the second-order steady-streaming velocity $v_{2}^{s}$ driven by a pressure difference Π(t)= (3/4)cos⁡(t)+(1/4)cos⁡(2t) in a canal with β=0.4 for α=5 and α=10. Fixed constant streamline spacings $\delta \psi_{2}$ are used for the plots, with $\delta \psi_{2}=5\cdot 10^{-4}$ for α=5 and $\delta \psi_{2}=3\cdot 10^{-3}$ for α=10.

The integral expressions (41)–(43) can be used to evaluate the corrections to the streaming velocity $v_{2}^{s}=\left(u_{2}^{s},v_{2}^{s}\right)$ arising at order $\varepsilon^{2}$. The solution was computed for different values of the channel undulation β and the Womersley number. Sample streamlines and associated vorticity contours are shown in Fig. 4 for β=0.4 and two values of α. The solution, symmetric about x=0.5, exhibits open streamlines aligned with the channel, whose walls delineate a stream tube carrying a nonzero flow rate. The flow structure is complicated by the presence of vortices with alternating circulation. For increasing α, these vortices approach the section of minimum width, with the velocity profile across the section of maximum width developing a nearly parabolic shape.

**Fig. 5.**
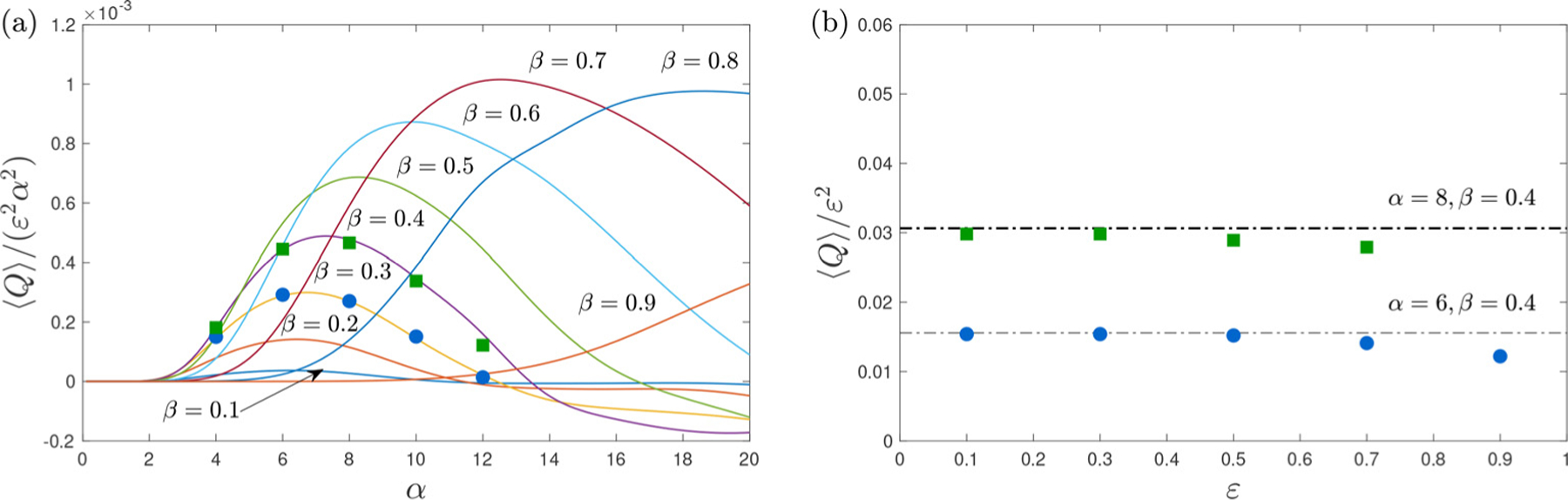
The mean flow rate ⟨Q⟩ as obtained from the asymprotic analysis for ε&1 (curves) and from the DNS computations in a channel with $h_{a}/\lambda =1/20$ (symbols). The plors show (a) the variation of $\langle Q\rangle /\left(\varepsilon^{2}\alpha^{2}\right)$ for increasing α and different values of β (DNS results computed with =0.1) and (b) a comparison for β=0.4 of the asymprotic predictions $\left\langle Q\rangle /\varepsilon^{2}=0.0306(\alpha =8\right)$ and $\left\langle Q\rangle /\varepsilon^{2}=0.0156(\alpha =6\right)$ with the values obtained numerically for increasing values of ε.

The variation with β and α of the net flow rate induced by the non-sinusoidal pressure difference of Fig. 2(a) was evaluated with use made of (44), giving the results shown in Fig. 5(a). The dependence on $\alpha^{2}$ is seen to be non-monotonic, with ⟨Q⟩ increasing initially to reach a maximum at an intermediate value of α, beyond which ⟨Q⟩ decreases to eventually become negative. The dependence on β also is non-monotonic, with ⟨Q⟩ vanishing in the two limiting cases β≪1, when the velocity approaches the Womersley solution with negligible convective acceleration, and 1−β≪1, when the pronounced wall undulation strangles the flow. According to the figure, values of in the range 0.7<β<0.8 result in the largest streaming motion for the specific periodic pressure considered in the computation.

### 4.2. Comparisons with direct numerical simulations

In order to validate the present theoretical model and assess its associated accuracy, the analytic predictions were compared with results of direct numerical simulations (DNS) employing the complete Navier-Stokes Eqs. (2)–(4) to describe oscillatory flow with finite values of the stroke length ε in a slender channel with $h_{0}/\lambda =1/20$ and total length 3λ. The computations were carried out with the finite-volume solver Ansys Fluent (Release 20.2), assuring second-order accuracy in time and in space. A coupled algorithm was used for the pressure-velocity coupling. The computational domain was discretized using a structured uniform mesh. A grid sensitivity analysis was conducted to ensure the grid-size independence of the results. To that aim, a mesh initially containing 9×10^4^ cells was systematically refined until no variation was obtained for the mean flow rate ⟨Q⟩, in a channel with β=0.3 and β=0.4 for α=(6,8,10), resulting in a final configuration containing a total of 6×10^5^ cells, which was used in all computations presented below. While the numerical evaluation of the integrals involved in the theoretical predictions takes seconds on a laptop computer, the DNS computations are significantly more expensive, with each simulation taking more than 60 h of CPU time to run 150 cycles, as needed to ensure convergence to a periodic solution with negligible inter-cycle variation, in a computational cluster using a total of 24 cores.

The converged numerical solution was used to compute the streaming flow by time-averaging the periodic velocity. Predicted mean flow rates $\langle Q\rangle =\int_{0}^{H}\;\langle u\rangle \text{d}y$ for ε=0.1 and different values of α and β are represented as symbols in Fig. 5(a). As can be seen, the departures of the asymptotic predictions from the DNS results are very small for the conditions explored in the figure, thereby validating the theoretical development. Additional numerical results obtained for β=0.4 and increasing values of ε are compared in Fig. 5(b) with the asymptotic predictions $\left\langle Q\rangle /\varepsilon^{2}=0.016(\alpha =6\right)$ and $\left\langle Q\rangle /\varepsilon^{2}=0.031(\alpha =8\right)$. The comparison reveals that the analytic results, which are strictly valid for asymptotically small values of ε≪1, remain reasonably accurate for values of the stroke length exceeding ε=0.5. This unexpected extended validity is consistent with previous findings pertaining to oscillating flow around circular cylinders [24].

**Fig. 6.**
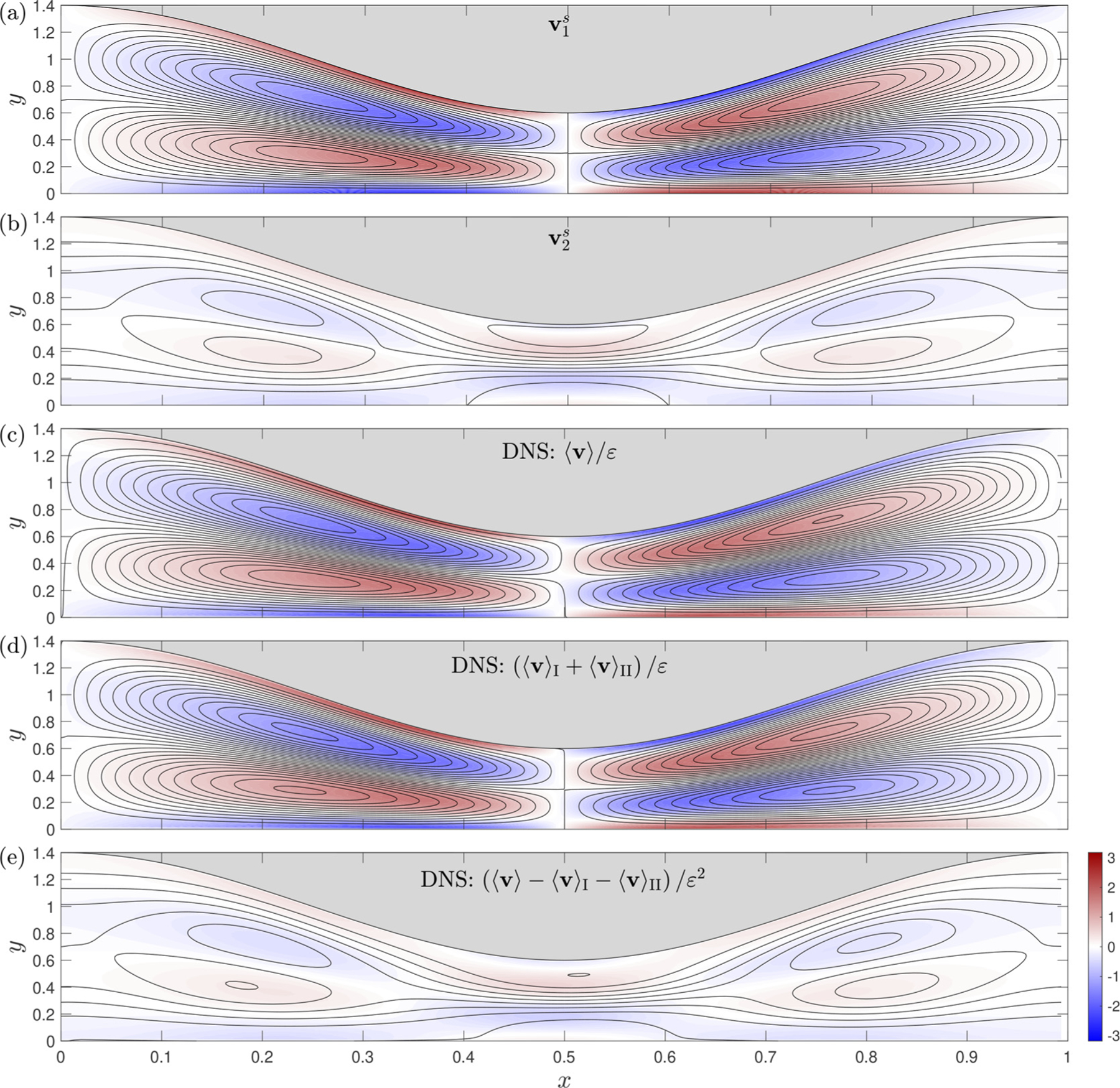
Streamlines and colour contours of vorticity corresponding to time-averaged flow in a channel with β=0.4 and α=6. The top rwo panels correspond to results of the asymptocic analysis for Π(r)=3/4cos⁡(τ)+1/4cos⁡(2τ), including (a) the first-order steady-screaming velocity $v_{1}^{5}$ and (b) the second-order steady-streaming velocity $v_{2}^{s}$. The botrom three panels correspond to DNS results with ε=0.1 and $h_{\sigma }/\lambda =1/20$. For comparison with the asymprotic results, the time-averaged velocities ⟨v⟩. $\langle v{)}_{\text{I}}$, and $\langle v\rangle_{1}$. obtained with Π(τ)=(3/4)cos⁡(t)+(1/4)cos⁡(2t),Π(t)=(3/4)cos⁡(t) and Π(c)=(1/4)cos⁡(L), respectively, are represented in the rescaled form (c)⟨v⟩/ε, (d) $\left(\langle v\rangle_{1}+\langle v\rangle_{\Pi 1}\right)/\varepsilon$, and $(\text{e})\left(\langle v\rangle -\langle v\rangle_{\text{I}}-\langle v\rangle_{∥1}\right)/\varepsilon^{2}$, A fixed constant streamline spacing $\delta \psi =2\cdot 10^{-2}$ is used for all plors.

Additional comparisons between the DNS and the theoretical predictions are shown in Fig. 6 for a channel with β=0.4 and α=6. Fig. 6(a) and (b) correspond to the steady-streaming velocity $v_{1}^{s}=\left(u_{1}^{s},v_{1}^{s}\right)$ and its correction $v_{2}^{s}=\left(u_{2}^{s},v_{2}^{s}\right)$ as obtained from the asymptotic analysis at order ε and $\varepsilon^{2}$, respectively, using the driving pressure Π(t)=(3/4)cos⁡(t)+ (1/4)cos⁡(2t) shown in Fig. 2(a). The corresponding time-averaged velocity distribution obtained in DNS computations with ε=0.1 is shown in Fig. 6(c). As can be seen in this last figure, the flow structure exhibits a slight aft-and-fore asymmetry, which is attributable to the multi-frequency character of the driving pressure. To further explore this issue, separate DNS computations were performed for the two sinusoidal signals Π(t)=(3/4)cos⁡(t) and Π(t)=(1/4)cos⁡(2t), with corresponding time-averaged velocities denoted by $\langle v\rangle_{\text{I}}$ and $\langle v\rangle_{\text{II}}$, respectively. The sum of these two contributions, appropriately scaled with ε, is plotted in Fig. 6(d). The resulting symmetric flow structure is to be compared with the leading-order asymptotic prediction $v_{1}^{s}$ shown in Fig. 6 (a). The results are nearly identical, with the peak stream function at the center of the vortices differing by less than 4%.

The observed differences between the DNS results in Fig. 6(c) and (d) are a consequence of the inter-mode interactions, which are responsible for the generation of the streamwise mean flow, described by the velocity function $v_{2}^{s}=\left(u_{2}^{s},v_{2}^{s}\right)$ in the asymptotic analysis at order $\varepsilon^{2}$. These inter-mode interactions were quantified from the DNS results by plotting $\langle v\rangle -\langle v\rangle_{\text{I}}-\langle v\rangle_{\text{II}}$ in Fig. 6(e), with the scale $\varepsilon^{2}$ introduced for comparison with the velocity field $v_{2}^{s}$ shown in Fig. 6(b). The small differences observed between the steady streaming resulting from intermode-interactions computed numerically (i.e. ($\langle v\rangle -\left.\langle v\rangle_{\text{I}}-\langle v\rangle_{\text{II}}\right)/\varepsilon^{2}$) and that predicted by the asymptotic analysis (i.e. $v_{2}^{s}$), on the order of 10%, are compatible with the relative errors of order ε expected in the asymptotic description. Clearly, the satisfactory agreement displayed in Fig. 6 between the DNS results and the theoretical predictions provide additional confidence in the latter.

### 4.3. Cardiac and respiratory wave forms

The streaming flow correction $v_{2}^{s}=\left(u_{2}^{s},v_{2}^{s}\right)$, identically zero in the sinusoidal case, depends fundamentally on the interactions of the different Fourier modes that define the shape of the periodic driving pressure Π(t). In the asymptotic analysis, these interactions, which are apparent in (23), lead to the forcing term ℱ that ultimately determines the streaming flow (41)–(44). Given the complexity of the associated algebra, it is not straightforward to ascertain how the interplay of the different modes dictates the final outcome. For example, for the two-mode pressure-wave form Π(t)= (3/4)cos⁡(t)+(1/4)cos⁡(2t), it was demonstrated in Fig. 5(a) that the resulting flow rate can be positive or negative depending on the value of α, but this finding might depend fundamentally on the pressure-wave form. Also, the linear dependence on $\alpha^{2}$ revealed in (44) appears to indicate that pressure signals with a large high-frequency content (and therefore a higher effective Womersley number) might lead to more pronounced effects, but this has to be tested by investigating different waveforms, as done below by using as illustrative example the oscillatory motion in the spinal canal.

**Fig. 7.**
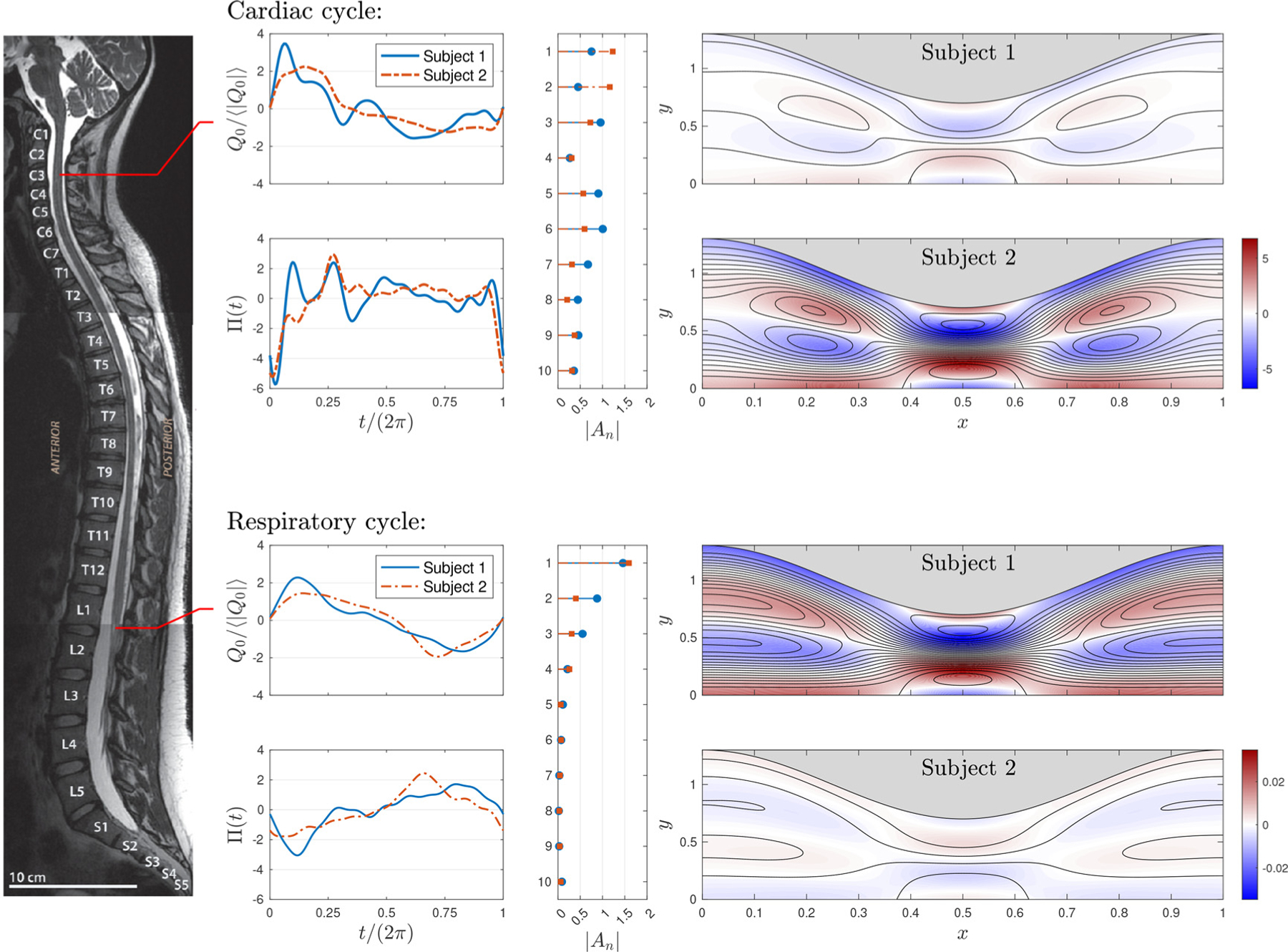
Temporal variation of the normalized flow rate $Q_{0}/\left\langle \left|Q_{0}\right|\right\rangle$ induced by the cardiac cycle in the cervical region [31] and by the respiratory cycle in the lumbar region [30] as obtained for two different healthy subjects from magnetic resonance measurements, The pressure signals Π and associated Fourier coefficients $A_{n}$ shown in the accompanying panels are determined using (19) for β=0.3 with α=6 (cardiac) and α=2.68 (respiratory), with the resulting signal normalized to give ⟨|Π|⟩=1. The panels on the right-hand side of the figure represent the streamlines and colour contours of vorticity corresponding to the second-order steady-streaming velocity $v_{2}^{s}$ driven in the wavy-walled channel by the four pressure signals Π. Fixed constant streamfunction increments $\delta \psi_{2}$ are used to draw the streamlines, with $\delta \psi_{2}=1\cdot 10^{-2}$ for cardiac-driven flow and $\delta \psi_{2}=5\cdot 10^{-5}$ for respiratory-driven flow.

The oscillating flow of CSF is driven by the cardiac and respiratory cycles [25,26]. The cardiac-driven motion, induced by the pressure fluctuations generated in the cranial cavity by the inflow/outflow of arterial blood, is more pronounced in the cervical region [27,28], whereas the respiratory-driven motion, induced by abdominal pressure changes, is more pronounced in the lumbar region [29,30]. The two periodic motions have very different periods, i.e. 2π/ω≃1s for the cardiac cycle and 2π/ω≃5s for the respiratory cycle. Using the kinematic viscosity of CSF $v=0.7\times 10^{-6}{\;\text{m}}^{2}/\text{s}$ along with $h_{0}≃1-3\;\text{mm}$ for the characteristic width of the spinal canal [31,32] yields corresponding Womersley numbers 3≲α≲9 for the cardiacdriven flow and 1.34≲α≲4.02 for the respiratory-driven flow. As revealed by magnetic resonance imaging (MRI), the resulting periodic motion is markedly non-sinusoidal, with typical normalized flow rates (instantaneous flow rate divided by its mean magnitude over a cycle) shown in Fig. 7. As indicated in the figure, the measurements of the cardiac-driven flow were taken at C3 level, whereas those of the respiratory-driven flow were taken at L1-L2 level, with results given for two different subjects.

In order to use the experimental measurements to investigate effects of complex waveforms, it is of interest to relate the shape of the oscillatory flow rate to the shape of the associated driving pressure difference (6). The computation of the coefficients $A_{n}$ involves the following steps. First, the flow-rate signal determined experimentally was normalized using its mean amplitude and the result was used to calculate the first 10 modes of the corresponding Fourier series $Q_{0}/\left\langle \left|Q_{0}\right|\right\rangle =R\left[\sum_{n=0}^{10}\;B_{n}e^{\text{i}\text{nt}}\right]$. The expression was then substituted into (19) to determine by inspection the corresponding Fourier coefficients of the pressure difference. The final values $A_{n}$ utilized in the computations were rescaled to give ⟨|Π|⟩=1, as is appropriate to enable comparisons between different waveforms. The resulting coefficients $A_{n}$ were computed for α=6 (cardiac cycle) and α=2.68 (respiratory cycle) with β=0.3. Corresponding Π(t) functions are shown in separate panels of Fig. 7 along with the magnitude of the different coefficients $\left|A_{n}\right|$. As can be seen, the pressure signal associated with the cardiac cycle exhibits pronounced peaks, also present in intracranial pressure measurements [12,33], whereas that of the respiratory cycle is more rounded. Correspondingly, the Fourier series of the cardiac-driven pressure signal shows a significant high-frequency content, which is not present in the respiratory-driven pressure, for which the higher-order amplitudes $\left|A_{5}\right|-\left|A_{10}\right|$ are negligibly small.

To investigate the influence of the pressure waveform on streaming, the four different pressure signals Π(t) shown in Fig. 7 were used to compute the flow in the canonical channel-flow configuration considered here. The resulting secondorder steady-streaming velocity $v_{2}^{s}=\left(u_{2}^{s},v_{2}^{s}\right)$ is shown in the four panels on the right-hand side of Fig. 7, where a different scale is used for the stream function and vorticity of the cardiac and respiratory plots, as needed to accommodate their dissimilar magnitudes. As expected, since the Womersley number of the cardiac flow is larger, the associated streaming flow is much more vigorous, in agreement with the trends identified in Fig. 5(a). The general flow structure is similar to that depicted in Fig. 4, with the outer recirculating vortices of the cardiac cycle located closer to the section of minimum width, as can be expected from the previous results. There exist non-negligible inter-subject variability, with the first subject exhibiting much smaller cardiac-driven velocities but much larger respiratory-driven velocities.

**Fig. 8.**
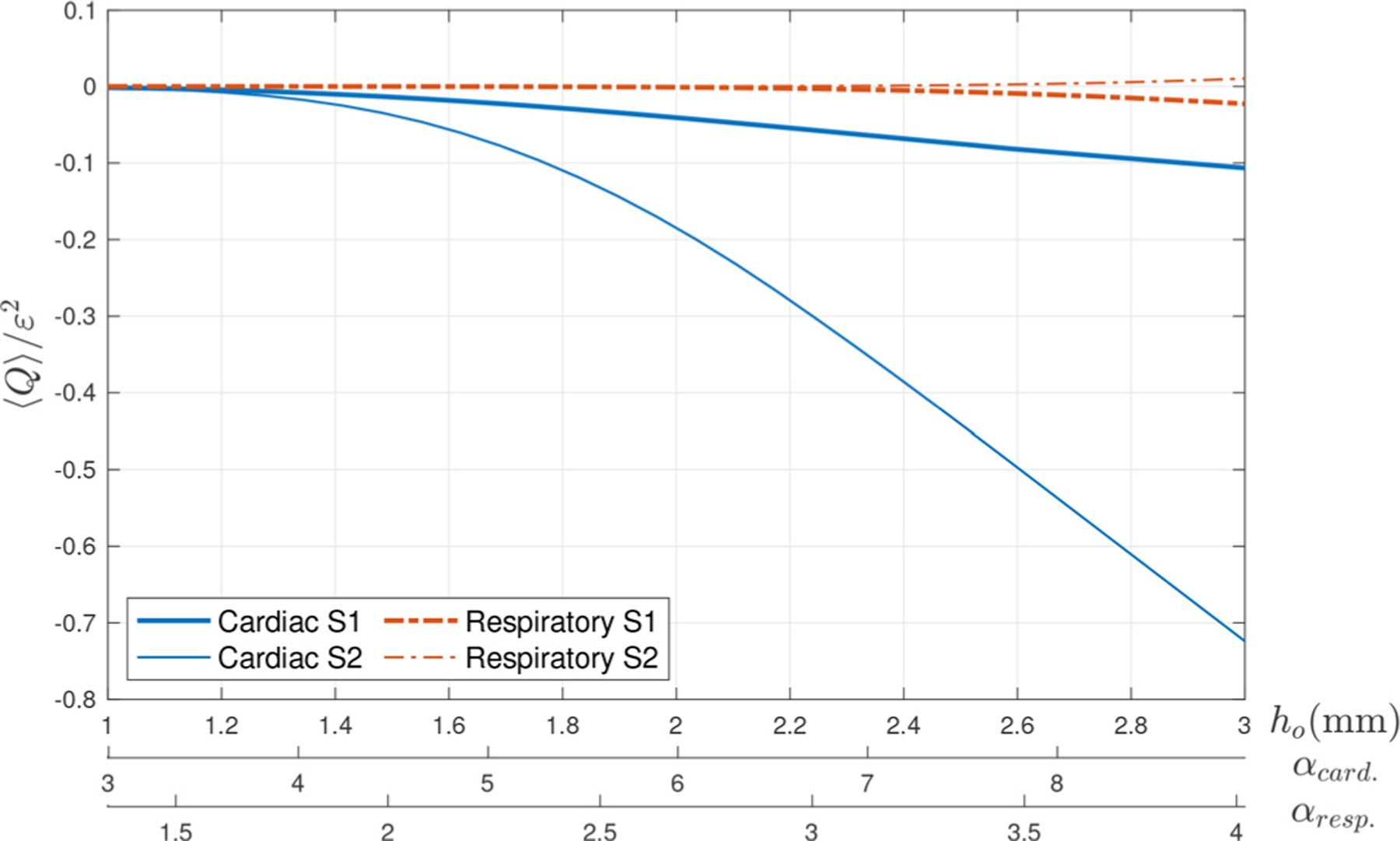
The variation with α of the rescaled mean flow rate $\langle Q\rangle /\varepsilon^{2}$ determined for β=0.3 using the cardiac and respiratory pressure signals derived from the normalized flow rates shown in Fig. 7.

For completeness, additional computations were carried out for different values of α using the pressure signals generated from the MRI flow-rate measurements, as delineated above. In the range of Womersley numbers explored, corresponding to a canal width $1\leq h_{0}\leq 3\;\text{mm}$ with $2\pi \omega^{-1}=1\;\text{s}$ (cardiac) and $2\pi \omega^{-1}=5\;\text{s}$ (respiratory), the magnitude of the flow rate increases monotonically, as shown in Fig. 8. The large differences in ⟨Q⟩ revealed by the figure further underline the intersubject variability previously mentioned. As can be seen, the respiratory-driven flow rate of subject 1 is negative, but that of subject 2 is positive, indicating that relative changes in the frequency distribution of the non-sinusoidal signal modify the streaming flow in nontrivial ways that are difficult to predict.

## 5. Conclusion

The steady streaming associated with a waveform with multiple harmonics, previously analyzed in the context of the flow induced by oscillating cylinders and spheres [17–19], has been investigated here for wall-bounded flows by asymptotic and numerical methods, with the two-dimensional wavy-walled channel used as canonical model configuration. A perturbation analysis in the limit of small stroke lengths ε≪1 provides closed-form analytic expression for the steady-streaming velocity $v_{1}^{s}=\left(u_{1}^{s},v_{1}^{s}\right)$ and its corrections $v_{2}^{s}=\left(u_{2}^{s},v_{2}^{s}\right)$. The latter, stemming from the interactions of the different Fourier modes that define the driving pressure gradient, carry a nonzero mean volumetric rate that breaks the fore-and-aft symmetry of the flow. The results of the asymptotic analysis are validated by comparisons with DNS computations. Somewhat unexpectedly, the asymptotic predictions for ε≪1 are found to remain quantitatively accurate for values of ε>0.5, well beyond the expected range of validity. Although a similar degree of agreement between asymptotic predictions and DNS computations has been found for ε~1 in other steady-streaming configurations, e.g. for flow about a linear array of cylinders [34], this result should be taken with caution, in that the relative departures can be dependent on the specific conditions. For instance, the agreement displayed in Fig. 5(b) might degrade for other values of α and β and also for pressure waveforms differing from that considered here, shown in Fig. 2(a).

The analysis indicates, in particular, that inter-frequency interactions leading to a net streamwise flow rate are more important for larger values of the Womersley number and intermediate values of the channel undulations. Sample computations using pressure gradients derived from in-vivo MRI measurements of the CSF flow rate in the spinal canal reveal large inter-subject variations. Also, the results suggest that the streaming flow resulting from inter-mode interactions is more pronounced for cardiac-driven flow, while it remains relatively small for respiratory-driven flow. Further steady-streaming studies addressing complex waveforms should account for the specific geometry of the spinal canal, thereby extending previous sinusoidal-pressure investigations [13,14,35]. These future studies should investigate, in particular, whether the additional steady-streaming motion arising from inter-mode interactions can modify significantly the spatial structure of the mean Lagrangian flow, possibly providing connectivity between the different closed recirculating regions that have been predicted to appear along the spinal canal [31] In this context, effects of microanatomical features, such as nerve roots and ligaments, not accounted for in the previous work [31], also warrant further investigation.

## Data Availability

Data will be made available on request.

## References

[1] Riley N, Steady streaming, Annu. Rev. Fluid Mech 33 (1) (2001) 43–65.

[2] Riley N, Oscillating viscous flows, Mathematika 12 (2) (1965) 161–175.

[3] Riley N, Oscillatory viscous flows. review and extension, IMA J. Appl. Math 3 (4) (1967) 419–434.

[4] Holtsmark J, Johnsen I, Sikkeland T, Skavlem S, Boundary layer flow near a cylindrical obstacle in an oscillating, incompressible fluid, J. Acoust. Soc. Am 26 (1) (1954) 26–39.

[5] Hall P, Unsteady viscous flow in a pipe of slowly varying cross-section, J. Fluid Mech 64 (2) (1974) 209–226.

[6] Grotberg JB, Volume-cycled oscillatory flow in a tapered channel, J. Fluid Mech 141 (1984) 249–264.

[7] Larrieu E, Hinch EJ, Charru F, Lagrangian drift near a wavy boundary in a viscous oscillating flow, J. Fluid Mech 630 (2009) 391–411.

[8] Nishimura T, Arakawa S, Murakami S, Kawamura Y, Oscillatory viscous flow in symmetric wavy-walled channels, Chem. Eng. Sci 44 (10) (1989) 2137–2148.

[9] Ralph M, Oscillatory flows in wavy-walled tubes, J. Fluid Mech 168 (1986) 515–540.

[10] Lo Jacono D, Plouraboué F, Bergeon A, Weak-inertial flow between two rough surfaces, Phys. Fluids 17 (6) (2005) 063602.

[11] Guibert R, Plouraboué F, Bergeon A, Steady streaming confined between three-dimensional wavy surfaces, J. Fluid Mech 657 (2010) 430–455.

[12] Singh V, Cheng R, Neurovascular physiology and neurocritical care, Handb. Clin. Neurol 176 (2021) 71–80. 33272411 10.1016/B978-0-444-64034-5.00014-6

[13] Sánchez AL, Martínez-Bazán C, Gutiérrez-Montes C, Criado-Hidalgo E, Pawlak G, Bradley W, Haughton V, Lasheras JC, On the bulk motion of the cerebrospinal fluid in the spinal canal, J. Fluid Mech 841 (2018) 203–227.

[14] Lawrence JJ, Coenen W, Sánchez AL, Pawlak G, Martínez-Bazán C, Haughton V, Lasheras JC, On the dispersion of a drug delivered intrathecally in the spinal canal, J. Fluid Mech 861 (2019) 679–720.

[15] Khani M, Sass LR, Xing T, Sharp MK, Balédent O, Martin BA, Anthropomorphic model of intrathecal cerebrospinal fluid dynamics within the spinal subarachnoid space: spinal cord nerve roots increase steady-streaming, J. Biomech. Eng 140 (8) (2018) 081012. 10.1115/1.4040401 PMC6056198 30003260

[16] Alaminos-Quesada J, Coenen W, Gutiérrez-Montes C, Sánchez AL, Buoyancy-modulated lagrangian drift in wavy-walled vertical channels as a model problem to understand drug dispersion in the spinal canal, J. Fluid Mech 949 (2022) A48. 37441053 10.1017/jfm.2022.799 PMC10338005

[17] Miyagi T, Nakahasi K, Secondary flow induced by an unharmonically oscillating circular cylinder, J. Phys. Soc. Jpn 39 (2) (1975) 519–526.

[18] Tatsuno M, Secondary flow induced by a circular cylinder performing unharmonic oscillations, J. Phys. Soc. Jpn 50 (1) (1981) 330–337.

[19] Higa M, Takahashi T, Stationary flow induced by an unharmonically oscillating sphere, J. Phys. Soc. Jpn 56 (5) (1987) 1703–1712.

[20] Davidson B, Riley N, Jets induced by oscillatory motion, J. Fluid Mech 53 (2) (1972) 287–303.

[21] Khani M, Sass LR, Sharp MK, McCabe AR, Zitella Verbick LM, Lad SP, Martin BA, In vitro and numerical simulation of blood removal from cerebrospinal fluid: comparison of lumbar drain to neurapheresis therapy, Fluids Barriers CNS 17 (1) (2020) 23. 32178689 10.1186/s12987-020-00185-5 PMC7077023

[22] Sass LR, Khani M, Romm J, Schmid Daners M, McCain K, Freeman T, Carter GT, Weeks DL, Petersen B, Aldred J, Wingett D, Martin BA, Non-invasive MRI quantification of cerebrospinal fluid dynamics in amyotrophic lateral sclerosis patients, Fluids Barriers CNS 17 (1) (2020) 4. 31959193 10.1186/s12987-019-0164-3 PMC6971921

[23] Hall P, Some unsteady viscous flows and their stability, ICST, 1973 Ph.D. thesis.

[24] Justesen P, A numerical study of oscillating flow around a circular cylinder, J. Fluid Mech 222 (1991) 157–196,

[25] Linninger AA, Tangen K, Hsu CY, Frim D, Cerebrospinal fluid mechanics and its coupling to cerebrovascular dynamics, Annu. Rev. Fluid Mech 48 (2016) 219–257.

[26] Kelley D, Thomas J, Cerebrospinal fluid flow, Annu. Rev. Fluid Mech 55 (1) (2023) 237–264. 10.1146/annurev-fluid-120720-011638 PMC11651633 39691763

[27] Tangen KM, Hsu Y, Zhu DC, Linninger AA, Cns wide simulation of flow resistance and drug transport due to spinal microanatomy, J. Biomech 48 (10) (2015) 2144–2154. 25888012 10.1016/j.jbiomech.2015.02.018

[28] Sincomb S, Coenen W, Gutiérrez-Montes C, Martínez Bazán C, Haughton V, Sánchez A, A one-dimensional model for the pulsating flow of cerebrospinal fluid in the spinal canal, J. Fluid Mech 939 (2022) A26. 36337071 10.1017/jfm.2022.215 PMC9635490

[29] Aktas G, Kollmeier JM, Joseph A, Merboldt K, Ludwig H, Gärtner J, Frahm J, Dreha-Kulaczewski S, Spinal CSF flow in response to forced thoracic and abdominal respiration, Fluids Barriers CNS 16 (10) (2019). 10.1186/s12987-019-0130-0 PMC6449937 30947716

[30] Gutiérrez-Montes C, Coenen W, Vidorreta M, Sincomb S, Martínez-Bazán C, Sánchez A, Haughton V, Effect of normal breathing on the movement of CSF in the spinal subarachnoid space, AJNR Am. J. Neuroradiol 43 (9) (2022) 1369–1374. 35981761 10.3174/ajnr.A7603 PMC9451622

[31] Coenen W, Gutiérrez-Montes C, Sincomb S, Criado-Hidalgo E, Wei K, King K, Haughton V, Martínez-Bazán C, Sánchez A, Lasheras JC, Subject-specific studies of CSF bulk flow patterns in the spinal canal: implications for the dispersion of solute particles in intrathecal drug delivery, AJNR Am. J. Neuroradiol 40 (7) (2019) 1242–1249. 31196863 10.3174/ajnr.A6097 PMC7048533

[32] Sass LR, Khani M, Natividad G, Tubbs RS, Baledent O, Martin BA, A 3D subject-specific model of the spinal subarachnoid space with anatomically realistic ventral and dorsal spinal cord nerve rootlets, Fluids Barriers CNS 14 (1) (2017) 36. 29258534 10.1186/s12987-017-0085-y PMC5738087

[33] Unnerbäck M, Ottesen J, Reinstrup P, ICP curve morphology and intracranial flow-volume changes: a simultaneous ICP and cine phase contrast MRI study in humans, Acta Neurochir. 160 (2) (2018) 219–224. 29273948 10.1007/s00701-017-3435-2 PMC5766711

[34] Alaminos-Quesada J, Lawrence J, Coenen W, Sánchez A, Oscillating viscous flow past a streamwise linear array of circular cylinders, J. Fluid Mech 959 (2023) A39. 37206991 10.1017/jfm.2023.178 PMC10191390

[35] Gutiérrez-Montes C, Coenen W, Lawrence JJ, Martínez-Bazán C, Sánchez AL, Lasheras JC, Modelling and direct numerical simulation of flow and solute dispersion in the spinal subarachnoid space, Appl. Math. Modelling 94 (2021) 516–533.

